# 1584. Retrospective Study Comparing the Rate of HIV Blips With Combined Antiretroviral Therapy

**DOI:** 10.1093/ofid/ofad500.1419

**Published:** 2023-11-27

**Authors:** Jennifer Makhoul, Surabhi Uppal, Anne A Barnes, Logan A Werner, Gary Simon, Marc O Siegel

**Affiliations:** UTH Houston/MD Anderson, Houston, Texas; Medstar Shah Medical Group, Oxon Hill, MD, washington, District of Columbia; George Washington University, Washington, District of Columbia; GW SMHS, merrick, New York; The George Washington University, Washington, DC; George Washington University School of Medicine and Health Sciences, Washington, District of Columbia

## Abstract

**Background:**

With the advancement of combined antiretroviral therapy (ART), control of viral replication has improved so that most patients living with HIV (PWH) can maintain undetectable viral. However, HIV blips (defined as non-sustained detectable low-level viremia) are still commonly encountered in practice. Although these generally do not indicate treatment failure or ART resistance, their clinical significance remains a topic of debate. The aim of our study is to compare the rate of blips in patients on bictegravir/emtricitabine/tenofovir alafenamide (BIC/FTC/TAF), rilpivirine/emtricitabine/tenofovir (RPV/FTC/TAF) or dolutegravir/abacavir/lamivudine (DOL/ABC/3TC).

**Methods:**

We analyzed HIV viral load (VL) data of PWH who were treated in our clinic with either BIC/FTC/TAF, RPV/FTC/TAF or DOL/ABC/3TC between 3/2017 and 10/2021 for blips. We defined low level blips as HIV VL of 51-200 copies/mL and high-level blips of201-1000 copies/mL. The incidence of blips between these regimens was examined using Chi squared test.

**Results:**

A total of 324 patients were included; 138 patients on BIC/FTC/TAF, 91 patients on RPV/FTC/TAF, and 95 patients on DOL/ABC/3TC). Patients on DOL/ABC/3TC were over twice as likely to have a viral blip (36%) compared to both BIC/FTC/TAF (16%, p=.0003) and RPV/FTC/TAF (14%, p=.0004). There were no statistically significant differences between patients taking BIC/FTC/TAF or RPV/FTC/TAF. Although the DOL/ABC/3TC group had a higher rate of high-level blips (6.3%) compared to BIC/FTC/TAF (3.6%) and RPV/FTC/TAF (4.4%), the total number of patients with high blips was low (n=15) and the differences were not statistically significant. Patients on DOL/ABC/3TC had average of 8 HIV VLs checked over the study period compared to 5 for patient on RPV/FTC/TAF and 3 for patient on BIC/FTC/TAF.

**Conclusion:**

Our data suggests that PWH on DOL/ABC/3TC have higher rates of blips compared to PWH on BIC/FTC/TAF or RPV/FTC/TAF. Additional studies are needed to determine if this might be due to the lack of tenofovir alafenamide in the DOL/ABC/3TC or due to increased HIV VL testing in this population.

**Disclosures:**

**All Authors**: No reported disclosures

